# Prognostic Impact of Multiple Synchronous T1 Breast Cancer

**DOI:** 10.3390/cancers16234019

**Published:** 2024-11-30

**Authors:** Hongki Gwak, Sung Hoo Jung, Young Jin Suh, Seok Jin Nam, Jai Hong Han, Se Jeong Oh, Eun Hwa Park, Seong Hwan Kim

**Affiliations:** 1 Division of Thyroid and Breast Surgical Oncology, Department of Surgery, Hwahong Hospital, Suwon 16630, Republic of Korea; hkgwak@gmail.com; 2 Division of Breast and Endocrine Surgery, Department of Surgery, Medical School, Chonbuk National University, Jeonju 54907, Republic of Korea; shjung@jbnu.ac.kr; 3 Division of Breast and Thyroid Surgical Oncology, Department of Surgery, The Catholic University of Korea, St. Vincent’s Hospital, Suwon 16247, Republic of Korea; yjsuh@catholic.ac.kr; 4 Division of Breast Surgery, Department of Surgery, Samsung Medical Center, School of Medicine, Sungkyunkwan University, Seoul 06351, Republic of Korea; sjnam@skku.edu; 5 Department of Surgery, Center for Breast Cancer, National Cancer Center, Goyang-si 10408, Republic of Korea; 13154@ncc.re.kr; 6 Department of Surgery, Incheon St. Mary’s Hospital, College of Medicine, The Catholic University of Korea, Seoul 06591, Republic of Korea; ohsj@catholic.ac.kr; 7 Department of Surgery, Dong-A University College of Medicine, Busan 49021, Republic of Korea; silversea75@nate.com; 8 Department of Plastic and Reconstructive Surgery, Kangnam Sacred Heart Hospital, Hallym University College of Medicine, Seoul 07441, Republic of Korea

**Keywords:** breast cancer, multiple tumors, overall survival, breast cancer-specific survival, T1 stage, escalated treatment

## Abstract

**Background/Objectives**: The reported incidence of multiple breast cancers varies widely, ranging from 6 to 60%, depending on the definitions used and methods of detection. With advancements in preoperative imaging techniques, such as magnetic resonance imaging, the detection of multiple breast cancers has improved. However, the clinical significance of multiple breast cancers remains controversial, with conflicting results regarding their impact on prognosis. We investigated the association between the number of synchronous ipsilateral T1 breast tumors, overall survival (OS), and breast cancer-specific survival (BCSS). **Methods**: We retrospectively analyzed 45,881 patients diagnosed with invasive breast cancer who underwent surgery between 2004 and 2016. The patients were categorized based on the number of tumors: one (*n* = 43,234), two (*n* = 2241), and three or more (*n* = 406). The OS and BCSS scores were compared across the groups. **Results**: There were no significant differences between the one- and two-tumor groups (*p* = 0.490 and *p* = 0.650, respectively). However, patients with three or more tumors had significantly lower OS and BCSS rates than those with one or two tumors (*p* < 0.001 for both comparisons). Multivariate analysis confirmed that the number of tumors (three or more) was an independent risk factor for poor OS and BCSS. **Conclusions**: Our findings suggest that patients with synchronous ipsilateral T1 breast cancers and three or more tumors may benefit from escalated treatment strategies due to their potentially worse prognosis.

## Simple Summary

This study investigated the association between the number of synchronous ipsilateral T1 breast tumors and patient survival. A retrospective analysis of 45,881 patients with invasive breast cancer was conducted. Patients were categorized based on the number of tumors: one, two, or three or more. Overall survival (OS) and breast cancer-specific survival (BCSS) were compared across groups. Patients with three or more tumors had significantly lower OS and BCSS rates compared to those with one or two tumors. Multivariate analysis confirmed the number of tumors as an independent risk factor for poor prognosis. Our findings suggest that patients with three or more synchronous ipsilateral T1 breast tumors may benefit from escalated treatment strategies due to their increased risk of mortality.

## 1. Introduction

Multiple breast cancers are classified into multifocal breast cancer, which refers to the presence of more than one tumor focus within the same quadrant of the breast, or multicentric breast cancer, which refers to the presence of more than one tumor focus in different quadrants of the breast [1]. However, owing to the lack of consistency in the criteria for classification and anatomical ambiguity, it is referred to as “multiple breast cancer” [2]. The incidence of multiple breast cancers varies widely in the literature, ranging from 6% to 60%, depending on the definitions and methods of detection used [3]. With advancements in preoperative imaging techniques, such as magnetic resonance imaging, the detection of multiple breast cancers has improved [4].

The clinical implications of multiple breast cancers are not well established. Some studies suggest that multiple breast cancer is associated with a higher tumor stage, a higher grade, lympho-vascular invasion, lymph node metastasis, and worse survival outcomes than unifocal breast cancer [1,3,5,6,7,8,9]. However, other studies report no significant differences in these parameters or prognostic factors between multiple and single breast cancers [10,11]. The existing TNM staging system does not consider the number of tumor foci or their location and considers only the size of the largest lesion to assign the T stage [12].

T1 breast cancer measures ≤ 2 cm in its greatest dimension [2]. It is considered an early-stage breast cancer with a favorable prognosis and a low risk of recurrence. However, T1 breast cancer can also be multifocal or multicentric, and the effect of multiple tumors on prognosis and management remains unclear [13].

The size of breast cancer is an important predictor of axillary metastasis and has a significant impact on prognosis. As the number of tumors increases, the tumor burden increases. However, according to the staging guidelines, the T stage is determined by the size of the largest mass in synchronous multiple breast cancer [2]. Therefore, it is highly likely that the tumor burden is underestimated in multiple breast cancers, especially T1 breast cancer, owing to small tumor size. Therefore, we evaluated the effect of tumor burden on prognosis according to the number of tumors in T1 breast cancer.

## 2. Materials and Methods

### 2.1. Patients

**Figure 1:**
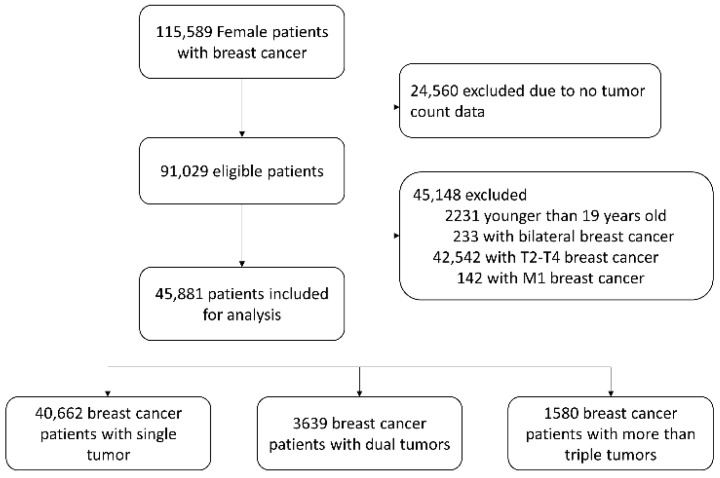
Flow diagram of patient selection.

This study was conducted using nationwide multicenter data prospectively collected by the Korean Breast Cancer Society from 102 general hospitals in South Korea [14]. Treatment and follow-up of breast cancer patients adhered to the Korean breast cancer clinical practice guidelines, which were developed based on the NCCN guidelines [15]. We selected adult females with stage T1 cancer who had undergone surgery for invasive breast cancer between January 2004 and December 2016. Patients with bilateral breast cancer or distant metastases were excluded (Figure 1).

The data included patient age, TNM stage, number of tumors, size of the largest tumor, histological classification, surgical method, estrogen receptor (ER) status, human epidermal growth factor 2 (HER2) status, diagnostic date, and the date of death. HER2 positivity was defined as either 3+ overexpression in immunohistochemical staining or HER2 amplification in fluorescent in situ hybridization (HER2/CEP17 ratio ≥ 2.0). Hormone receptor positivity was defined as >1% staining for either ER or progesterone receptor or both. Human epidermal growth factor receptor 2 (HER2) positivity was defined as either 3+ overexpression or HER2 amplification observed via immunohistochemical staining or fluorescent in situ hybridization, respectively (HER2/chromosome enumeration probe 17 ratios: ≥2.0). Hormone receptor (HR) positivity was defined as >1% staining for either estrogen or progesterone receptors or both.

### 2.2. Statistical Analyses

Patients were classified into three groups based on the number of tumors: one, two, or three or more. Their clinical characteristics were compared using an analysis of variance and Student’s *t*-test for continuous and non-continuous variables, respectively. Survival was defined as the interval between the date of diagnosis and the date of death. Survival was estimated using the Kaplan–Meier method and compared using the restricted mean survival time (RMST) and log-rank test (two-sided, *p* < 0.05). Cox proportional regression models were used to estimate the hazard ratios (95% confidence intervals [CIs]) for breast cancer-specific survival (BCSS) and overall survival (OS). Propensity score matching (PSM) was performed using the MatchIt package. For improving study power, 1:4 nearest neighbor matching without replacement was used with propensity scores estimated through logistic regression [16]. Statistical analyses were performed using the R software (ver. 4.4.1, R Core Team, 2024, Vienna, Austria).

## 3. Results

### 3.1. Patient Characteristics

A total of 45,881 patients with invasive breast cancer were included in the analysis. Among them, 88.6% (*n* = 40,662) had a single tumor, 7.9% (*n* = 3639) had two tumors, and 3.4% had three or more tumors (*n* = 1580). The average size of the largest tumor was 1.24 cm, which did not differ among the three groups (*p* = 0.999; Table 1).

There was a significant positive correlation between the number of tumors and the proportion of patients aged 50 or younger (54.7% with one tumor, 62.5% with two, and 67.1% with three or more; *p* < 0.001). Additionally, a higher proportion of patients underwent mastectomy rather than breast-conserving surgery as the number of tumors increased (29.2% with one tumor, 50.7% with two, and 64.9% with three or more; *p* < 0.001).

### 3.2. Clinicopathological Characteristics

As the number of tumors increased, the proportion of patients with lobular carcinoma and HER2-positive breast cancer increased significantly. In patients with one, two, or three or more tumors, the incidence of axillary metastasis was 23.4%, 27.6%, and 33.5%, respectively (*p* < 0.001). Patients with a higher tumor count were significantly more likely to receive chemotherapy (55.4% with one tumor, 60.0% with two, and 65.2% with three or more; *p* < 0.001). Patient characteristics are summarized in Table 1.

### 3.3. Survival Analysis

**Figure 2:**
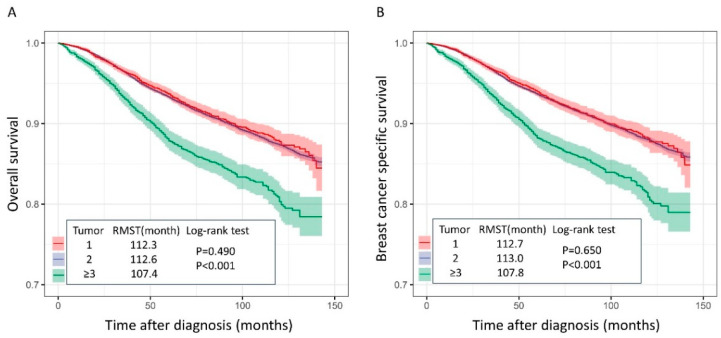
Kaplan–Meier curves for (**A**) overall survival and (**B**) breast cancer-specific survival.

The median follow-up duration was 62.4 (±40.3) months. The log-rank test results showed no significant differences in OS and BCSS between the one- and two-tumor groups (log-rank test *p* = 0.490, *p* = 0.650). However, OS and BCSS were significantly lower in the group with three or more tumors than in the groups with one and two tumors (*p* < 0.001; Figure 2).

The RMST for OS was not significantly different between the one- (112.3 ± 0.1 months) and two-tumor groups (112.6 ± 0.3 months) (*p* = 0.299). However, the RMST for OS was significantly shorter in the group with three or more tumors (107.4 ± 0.6 months) than in the other groups (*p* < 0.001). Similarly, the RMST for BCSS did not differ significantly between the patients in the one- (112.7 ± 0.1 months) and two-tumor groups (113.0 ± 0.3 months) (*p* = 0.309). However, the RMST for BCSS was significantly shorter in the patients in the group with three or more tumors (107.8 ± 0.6 months) than in those in the other groups (*p* < 0.001; Figure 2).

Multivariate Cox proportional analysis showed that OS and BCSS were lower in patients with three or more tumors than in those in the other groups (OS 1.386 (1.096–1.754), *p* = 0.006; BCSS 1.349 (1.054–1.727), *p* = 0.017; Table 2 and Table 3).

To mitigate the confounding effects of intergroup differences, we employed 1:4 PSM, effectively eliminating all significant between-group disparities (Table 4).

**Figure 3:**
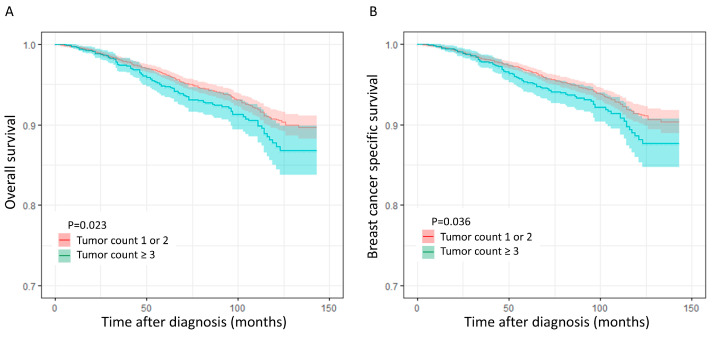
Kaplan–Meier curves after propensity score matching, (**A**) overall survival, and (**B**) breast cancer-specific survival.

Figure 3 presents a Kaplan–Meier (KM) curve comparing the survival rates between patients with two or fewer tumors and those with three or more tumors after propensity score matching (PSM) was applied to adjust for potential biases. Subsequent log-rank tests following PSM revealed that the patients in the group with three or more tumors exhibited significantly inferior OS and BCSS compared with those in the groups with one or two tumors (OS; *p* = 0.023, BCSS; *p* = 0.036; Figure 3).

## 4. Discussion

Breast cancer is a heterogeneous disease with various histological and molecular features and is divided into three major subtypes depending on the presence or absence of molecular markers: ER or progesterone receptor and HER2 [17]. Because the response to and prognosis of chemotherapy for breast cancer vary depending on the subtype, the revised TNM prognostic staging reflects histological and molecular characteristics [2,12,14].

We focused exclusively on patients with T1 breast cancer. Notably, 11.3% of patients presented with multifocal disease, which aligns with the findings of a broader meta-analysis encompassing various tumor stages [4]. Interestingly, no significant difference in the largest tumor size was observed across the groups categorized according to the number of tumors. These findings suggest that the number of tumors may not directly correlate with the size of the largest tumor. Therefore, clinicians should maintain a high index of suspicion of multifocal breast cancer, irrespective of the size of the dominant lesion.

Multiple breast cancer is associated with a poorer prognosis compared with single breast cancer [1,5,6,18,19]. However, some studies have reported no significant difference in prognosis between multiple breast cancer and single breast cancer [10,11]. The effect of lymph node metastasis on the prognosis of multiple breast cancer remains controversial [20]. These studies often employed a binary classification (single vs. multiple tumors), which may be overly simplistic in capturing the true extent of the tumor burden. Although the sum of tumor diameters has been used to quantify tumor burden in multiple breast cancers, this approach may lack intuitiveness and ease of calculation [7,8]. To address these limitations, a straightforward and easily implemented method is required. We aimed to address this limitation by analyzing the relationship between prognosis and the number of tumors, a readily obtainable metric in clinical practice.

In the TNM staging system, the T stage relies solely on the size of the largest tumor for disease classification and treatment planning. However, this approach may not adequately capture the total tumor burden in patients with multifocal breast cancer, potentially underestimating disease aggressiveness. Our results align with the previous findings of Nathan et al. [8]. We observed a higher prevalence of axillary lymph node metastasis in patients with multiple breast cancers than in those with a single tumor. The rate of metastasis correlated positively with the number of tumors.

Interestingly, our study revealed no significant differences in OS or BCSS between patients with single and dual tumors. However, a statistically significant difference in survival rates emerged in patients with three or more tumors. These findings suggest a potential threshold effect in which tumor burden alone is a significant prognostic factor for three or more lesions. This may partially explain the heterogeneous results reported in other studies investigating the prognosis of multiple breast cancers.

The heterogeneous nature of tumors in patients with multiple breast cancer is another factor that contributes to the inconsistent prognostic outcomes observed in this patient population. Each tumor in a patient with multiple breast cancer may exhibit a distinct histological subtype, potentially influencing treatment response and OS. Buggi et al. reported that ER status was discordant in 4.4%, PR status was discordant in 15.9%, and HER2 status was discordant in 9.7% of patients with multiple breast cancers, resulting in a total of 12.4% of patients not receiving the correct adjuvant treatment due to heterogeneity [21]. Pekar et al. reported that 10% to 12.7% of women with ipsilateral multiple synchronous breast cancers had a heterogeneous subtype, and patients with a heterogeneous subtype had significantly worse survival [22].

Although our findings provide valuable insights into the prognostic implications of tumor burden in multiple breast cancers, it is important to acknowledge the limitations. First, the absence of molecular pathological data on individual tumors impedes a more comprehensive evaluation of tumor heterogeneity and its potential impact on prognosis. Future research incorporating the molecular characterization of each tumor could refine our understanding of this complex relationship. Second, the retrospective nature of our study limited our ability to draw definitive causal inferences. Prospective studies with rigorously controlled designs would be better suited to establish causal relationships between tumor burden and prognosis. Thirdly, the absence of information regarding BRCA germline mutations is noteworthy. While BRCA mutations are well-established risk factors for breast cancer, particularly bilateral disease, their association with ipsilateral multiple breast cancer remains unclear [23]. The prognostic impact of BRCA mutations on ipsilateral multiple breast cancer remains controversial, despite the majority of studies suggesting a worse outcome for BRCA-mutated breast cancer [24,25,26,27,28,29,30,31].

Despite these limitations, our study has several strengths. This is the first study to investigate the prognostic impact of tumor count in patients with T1 breast cancer utilizing a national database that encompasses a large and representative sample of patients with multiple breast cancers. We included ER and HER2 statuses, providing a more comprehensive assessment of prognostic factors than assessments limited to ER analysis [4].

## 5. Conclusions

Our findings suggest that tumor burden may be a significant prognostic factor for multiple breast cancers, particularly in patients with three or more tumors. These findings may inform treatment decisions, potentially favoring escalated therapeutic approaches for patients with a higher tumor burden. However, further research is needed to address these limitations, particularly regarding tumor heterogeneity. Future studies incorporating molecular analyses and rigorous classification criteria could definitively establish the role of these factors in predicting prognoses and guiding personalized treatment strategies in patients with multiple breast cancers.

## Figures and Tables

**Table 1:** Clinicopathological characteristics.

	Number of Tumors (*n* = 45,881)			
	One (*n* = 40,662)	Two (*n* = 3639)	Three or More (*n* = 1580)	*p*-Value
Age, years				<0.001
<30	378 (0.9)	30 (0.8)	19 (1.2)	
30–50	21,861 (53.8)	2245 (61.7)	1041 (65.9)	
≥50	18,423 (45.3)	1364 (37.5)	520 (32.9)	
Largest tumor size (cm)	1.243 ± 1.048	1.244 ± 0.681	1.243 ± 0.766	0.999
Surgery				<0.001
Breast-conserving	28,784 (70.8)	1793 (49.3)	554 (35.1)	
Mastectomy	11,878 (29.2)	1846 (50.7)	1026 (64.9)	
N stage				<0.001
N0	31136 (76.7)	2635 (72.6)	1052 (66.8)	
N1	7956 (19.6)	833 (22.9)	403 (25.6)	
N2	1079 (2.7)	109 (3.0)	76 (4.8)	
N3	429 (1.1)	53 (1.5)	44 (2.8)	
Unknown	62	9	5	
Hormonal receptor				<0.001
Positive	30,942 (76.2)	2900 (79.8)	1239 (78.6)	
Negative	9667 (23.8)	735 (20.2)	337 (21.4)	
Unknown	53	4	4	
HER2				<0.001
Positive	11,346 (27.9)	1086 (29.9)	554 (35.2)	
Negative	29,263 (72.1)	2549 (70.1)	1022 (64.8)	
Unknown	53	4	4	
Chemotherapy				<0.001
No	18097 (44.6)	1452 (40.0)	549 (34.8)	
Yes	22505 (55.4)	2179 (60.0)	1029 (65.2)	
Unknown	60	8	2	

**Table 2:** Univariate and multivariate analyses of prognostic factors for overall survival.

	Univariate Analysis	*p*-Value	Multivariate Analysis	*p*-Value
Age, years				
<30	1		1	
30–50	0.620 (0.526–0.732)	<0.001	0.575 (0.382–0.865)	0.008
≥50	0.909 (0.771–1.072)	0.257	0.909 (0.606–1.365)	0.647
Surgery				
Breast-conserving	1		1	
Mastectomy	2.336 (2.231–2.445)	<0.001	1.946 (1.829–2.071)	<0.001
Tumor type				
IDC	1		1	
ILC	0.848 (0.730–0.985)	0.031	1.197 (0.832–1.723)	0.332
Others	1.109 (0.967–1.271)	0.139	1.264 (0.821–1.946)	0.281
Axillary metastasis				
No	1		1	
Yes	3.829 (3.656–4.010)	<0.001	2.635 (2.377–2.922)	<0.001
Tumor count				
1	1		1	
2	0.959 (0.802–1.145)	0.640	0.937 (0.768–1.144)	0.525
≥3	1.489 (1.218–1.822)	<0.001	1.386 (1.096–1.754)	0.006
Molecular subtype				
Luminal A	1		1	
Luminal B	1.648 (0.455–0.550)	<0.001	1.355 (1.110–1.655)	0.003
Luminal HER2	2.312 (2.049–2.607)	<0.001	2.139 (1.707–2.681)	<0.001
HER2	2.806 (2.490–3.162)	<0.001	2.492 (1.988–3.125)	<0.001
TNBC	3.651 (3.271–4.075)	<0.001	3.414 (2.774–4.202)	<0.001

**Table 3:** Univariate and multivariate analyses of prognostic factors for breast cancer-specific survival.

	Univariate Analysis	*p*-Value	Multivariate Analysis	*p*-Value
Age, years				
<30	1		1	
30–50	0.610 (0.517–0.720)	<0.001	0.560 (0.372–0.843)	0.005
≥50	0.843 (0.714–0.995)	0.043	1.349 (0.603–0.924)	0.017
Surgery				
Breast-conserving	1		1	
Mastectomy	2.354 (2.246–2.467)	<0.001	1.947 (1.826–2.075)	<0.001
Tumor type				
IDC	1		1	
ILC	0.850 (0.729–0.991)	0.038	1.260 (0.865–1.837)	0.229
Others	1.087 (0.944–1.252)	0.248	1.258 (0.799–1.980)	0.321
Axillary metastasis				
No	1		1	
Yes	4.084 (3.893–4.285)	<0.001	2.818 (2.531–3.138)	<0.001
Tumor count				
1	1		1	
2	0.992 (0.826–1.192)	0.935	0.972 (0.792–1.192)	0.785
≥3	1.473 (1.192–1.821)	<0.001	1.349 (1.054–1.727)	0.017
Molecular subtype				
Luminal A	1		1	
Luminal B	1.654 (1.478–1.849)	<0.001	1.343 (1.085–1.663)	0.007
Luminal HER2	2.389 (2.109–2.706)	<0.001	2.342 (1.847–2.970)	<0.001
HER2	2.911 (2.573–3.293)	<0.001	2.719 (2.141–3.454)	<0.001
TNBC	3.789 (3.381–4.245)	<0.001	3.744 (3.005–4.664)	<0.001

**Table 4:** Clinicopathological characteristics after propensity score matching.

	One or Two (*n* = 6292)	Three or More (*n* = 1573)	*p*-Value
Age, years			0.966
<50	4226 (67.2)	1055 (67.1)	
≥50	18,423 (45.3)	518 (32.9)	
Surgery			1.000
Breast-conserving	2204 (35.0)	551 (35.0)	
Mastectomy	4088 (65.0)	1022 (65.0)	
Nodal involvement			1.000
No	4200 (66.8)	1050 (66.8)	
Yes	2092 (33.2)	523 (33.2)	
Unknown			
Hormonal receptor			1.000
Positive	1342 (21.3)	335 (21.3)	
Negative	4950 (78.7)	1238 (78.7)	
HER2			0.948
Positive	4072 (64.7)	1020 (64.8)	
Negative	2220 (35.3)	553 (35.2)	
Tumor type			0.980
IDC	5858 (93.1)	1463 (93.0)	
ILC	256 (4.1)	64 (4.1)	
Others	178 (2.8)	46 (2.9)	

IDC, invasive ductal carcinoma; ILC, invasive lobular carcinoma.

## Data Availability

The datasets generated during and/or analyzed during the current study are available from the corresponding author on reasonable request.
